# Disease-related microglia heterogeneity in the hippocampus of Alzheimer’s disease, dementia with Lewy bodies, and hippocampal sclerosis of aging

**DOI:** 10.1186/s40478-015-0209-z

**Published:** 2015-05-23

**Authors:** Adam D. Bachstetter, Linda J. Van Eldik, Frederick A. Schmitt, Janna H. Neltner, Eseosa T. Ighodaro, Scott J. Webster, Ela Patel, Erin L. Abner, Richard J, Kryscio, Peter T. Nelson

**Affiliations:** Sanders-Brown Center on Aging, University of Kentucky, 800 S. Limestone St, Lexington, KY USA; Department of Anatomy and Neurobiology, University of Kentucky, Lexington, KY USA; Department of Neurology, University of Kentucky, Lexington, KY USA; Department of Pathology and Laboratory Medicine, Division of Neuropathology, University of Kentucky, Lexington, KY USA; Department of Epidemiology, University of Kentucky, Lexington, KY USA; Department of Biostatistics, University of Kentucky, Lexington, KY USA; Department of Statistics, University of Kentucky, Lexington, KY USA

**Keywords:** Aging, Microglia activation, Mixed dementia, Neurodegeneration, Neuroinflammation, Neuropathology

## Abstract

**Introduction:**

Neuropathological, genetic, and biochemical studies have provided support for the hypothesis that microglia participate in Alzheimer’s disease (AD) pathogenesis. Despite the extensive characterization of AD microglia, there are still many unanswered questions, and little is known about microglial morphology in other common forms of age-related dementia: particularly, dementia with Lewy bodies (DLB) and hippocampal sclerosis of aging (HS-Aging). In addition, no prior studies have attempted to compare and contrast the microglia morphology in the hippocampus of various neurodegenerative conditions.

**Results:**

Here we studied cases with pathologically-confirmed AD (*n* = 7), HS-Aging (n = 7), AD + HS-aging (*n* = 4), DLB (*n* = 12), and normal (cognitively intact) controls (NC) (*n* = 9) from the University of Kentucky Alzheimer’s Disease Center autopsy cohort. We defined five microglia morphological phenotypes in the autopsy samples: ramified, hypertrophic, dystrophic, rod-shaped, and amoeboid. The Aperio ScanScope digital neuropathological tool was used along with two well-known microglial markers: IBA1 (a marker for both resting and activated microglia) and CD68 (a lysosomal marker in macrophages/microglia associated with phagocytic cells). Hippocampal staining analyses included studies of subregions within the hippocampal formation and nearby white matter. Using these tools and methods, we describe variation in microglial characteristics that show some degree of disease specificity, including, (1) increased microglia density and number in HS-aging and AD + HS-aging; (2) low microglia density in DLB; (3) increased number of dystrophic microglia in HS-aging; and (4) increased proportion of dystrophic to all microglia in DLB.

**Conclusions:**

We conclude that variations in morphologies among microglial cells, and cells of macrophage lineage, can help guide future work connecting neuroinflammatory mechanisms with specific neurodegenerative disease subtypes.

## Introduction

There is an increasing awareness that microglia may have a pathogenic role in neurodegenerative diseases. The discovery of genetic mutations in CD33 and TREM2 associated with the risk of developing Alzheimer’s disease (AD) [1–4] has heightened the interest in defining microglia physiology and pathology in the context of disease. Pio Del Rio-Hortega is credited with early insights into microglial pathology. He recognized that microglia are normally highly ramified and evenly distributed throughout the brain. He also noted that the morphology of microglia is dramatically altered in response to central nervous system (CNS) pathology [5]. As a molecular and functionally unique population of cells [6, 7], microglia exhibit a remarkable ability to survey the brain and rapidly undergo a spectrum of responses to insults or tissue damage [8, 9]. The process by which microglia change shape, molecular signature, and cellular physiology is defined as microglia activation [5].

The clinical disease formerly referred to simply as “Alzheimer’s disease” is, at the population level, a complex manifestation of many different brain conditions [10]. These age-related brain pathologies include AD (characterized by amyloid plaques and neurofibrillary tangles), as well as cerebrovascular disease, dementia with Lewy bodies (DLB), and hippocampal sclerosis of aging (HS-Aging) [11]. Although each of these disorders seems to have a distinct genetic, clinical, and pathological cluster of characteristics, to date there has not been characterization of the microglial responses in these conditions.

We sought to address questions related to microglial morphology in neurodegenerative disease tissue: 1) Is microglia pathology seen only in the presence of amyloid or tau pathology, or can it be seen in other age-related neurodegenerative diseases?; 2) Is there microglial regional heterogeneity in the hippocampus (for example, gray matter only)?; and, 3) Can digital neuropathological quantification detect differences in microglia activation in different neurodegenerative diseases? To address these questions, we queried well-characterized brain samples from the University of Kentucky Alzheimer’s Disease Center (UK-ADC) cohort. Specifically, brain tissue was analyzed, incorporating multiple disease conditions, using two antibodies that react with microglia. The CD68 antibody stains for a lysosomal-associated protein in macrophages/microglia and is associated with phagocytic cells [12, 13]. The IBA1 (ionized calcium binding adaptor molecule 1) antibody [14] is used widely as a pan marker for both resting and activated microglia. Using these two widely studied microglia markers, CD68 and IBA1, we defined microglia morphologies in the aged brain, including some features that show evidence of disease specificity.

## Materials and methods

### Human subjects

Tissue samples that contained the hippocampus were acquired from the UK-ADC biobank. Details of recruitment have been described previously [15]. Information including demographic and neuropathologic data is presented (Table 1). The included cases (*n* = 39) represented a convenience sample subdivided into groups as: NC, HS-aging, AD, AD + HS-aging, or DLB. Cases represented approximately age-matched sampling of the neuropathologically-defined diseases using the following criteria: AD (Braak > IV, high density of neocortical amyloid plaques); isocortical subtype of DLB; and HS-Aging (cell loss and gliosis out of proportion to plaques/tangle pathology, with TDP-43 pathology in the hippocampus).

**Table 1:** Cohort demographics and numbers

case	age at death	Final MMSE	sex	ApoE alleles	PMI (h)	Braak stage	CERAD plaque stage	diffuse plaques	neuritic plaques	NFTs	diffuse Lewy bodies
NC = non-demented control: mean age =86; mean MMSE = 30; Median Braak stage = 2; Median CERAD = 0											
1	81	30	M	3 / 3	2.17	2	0	0	0	0	0
2	91	30	M	n/a	n/a	2	0	0	0	1.4	0
3	86	30	M	3 / 3	2.17	3	0	0	0	4.6	0
4	93	30	F	3 / 3	2.25	2	0	0	0	0.25	0
5	84	30	M	2 / 3	3.25	0	0	n/a	n/a	n/a	0
6	85	30	M	3 / 3	2	3	1	2	0.5	8	0
7	84	30	F	3 / 3	2.42	0	0	0	0	0	0
8	92	30	F	2 / 3	3.25	3	2	5	0	0.75	0
9	81	30	M	3 / 4	2	2	2	2	10.5	1	0
HS = hippocampal sclerosis of aging: mean age =87; mean MMSE = 22.7; Median Braak stage = 2; Median CERAD = 0											
10	74	20	M	3 / 4	8	3	2	0	0	1.4	0
11	95	16	F	3 / 4	3.25	3	0	0	0	1	0
12	87	28	F	3 / 3	1.82	3	0	0	0	4	0
13	84	10	F	n/a	2.57	2	0	0	0	0	0
14	91	29	F	2 / 3	2.87	2	1	0	0	6.8	0
15	91	30	M	3 / 3	2.83	2	0	n/a	n/a	n/a	0
16	88	26	M	3 / 4	2.33	0	0	0	0	0	0
AD = Alzheimer’s disease: mean age =77; mean MMSE = 11; Median Braak stage = 6; Median CERAD = 3											
17	75	18	F	3 / 4	2.5	6	3	9	0.33	25	0
18	84	13	M	4 / 4	5.17	6	3	4	1.33	26.75	0
19	65	3	F	3 / 4	4.1	6	3	2.67	1.67	41	0
20	85	4	F	3 / 3	11.2	6	3	6.67	1.33	78.8	0
21	79	6	M	n/a	2.08	6	3	4.33	2.33	25.6	0
22	67	11	M	2 / 3	1.75	6	3	0.33	0	2.4	+
23	82	25	M	n/a	2.75	6	2	1.67	2.67	19.8	+
AD + HS: mean age =91; mean MMSE = 7.8; Median Braak stage = 6; Median CERAD = 3											
24	96	6	F	3 / 3	6.75	6	3	n/a	n/a	n/a	0
25	91	13	F	3 / 3	3	5	3	6.67	1.33	34	0
26	91	12	F	4 / 4	2.33	6	3	0	0	10.25	+
27	87	0	F	3 / 3	2.67	6	3	0	0	54.6	0
DLB = Dementia with Lewy bodies: mean age =80; mean MMSE = 17.25; Median Braak stage = 2; Median CERAD = 1											
28	65	9	M	4 / 4	9.5	2	3	2	7.67	2	+
29	61	18	M	3 / 3	2	2	0	0	0	9.6	+
30	85	2	F	2 / 3	2	2	3	4	6.67	3.6	+
31	85	27	M	3 / 3	11.2	2	1	0	0	0.8	+
32	89	27	M	3 / 3	2.42	2	0	0	0	8.5	+
33	92	27	F	3 / 4	2.42	2	1	0.67	0	2	+
34	68	11	M	3 / 4	3.75	2	2	0	0	0.4	+
35	81	15	M	3 / 3	2.42	2	2	1.5	2.5	0.25	+
36	78	9	M	3 / 3	2.5	1	0	0	0	2	+
37	81	26	M	3 / 3	5.77	1	1	0	0	0	+
38	97	21	M	2 / 3	3.5	1	0	0	0	1.8	+
39	78	15	M	3 / 4	n/a	1	1	3.5	0.5	0	+

Counts in the hippocampus of neurofibrillary tangles (NFTs), and amyloid plaques, without neurites (diffuse plaques), and with degenerating neurites (neuritic plaques) (see [42]). Abbreviations: Apo E apolipoprotein E; PMI = post-mortem interval; MMSE = mini-mental state examination; +, feature present; n/a = not available

### Immunostaining

Paraffin-embedded tissue sections were cut at 10-μm-thick. Immunohistochemical (IHC) began with microwave antigen retrieval for 6 min (power 8) using Trilogy buffer (Cell Marque; Rocklin, CA) for CD68 and Declere buffer (Cell Marque; Rocklin, CA) for IBA1. Sections were then placed in 3% H_2_O_2_ in methanol for 30 min. Following washes in distilled water, sections were blocked in 5% goat serum at room temperature for 1 h. Sections were incubated in primary antibodies IBA1 (rabbit polyclonal, 1:1,000 IHC, Wako); CD68 (clone KP1) (1:50 IHC, Dako) overnight at 4°C. A biotinylated secondary antibody (Vector Laboratories) was amplified using avidin-biotin substrate (ABC solution, Vector Laboratories catalog no. PK-6100), followed by color development in Nova Red (Vector Laboratories). Immunofluorescence (IF) staining was done following microwave antigen retrieval for 6 min (power 8) using Declere buffer (Cell Marque; Rocklin, CA) for primary antibodies to: IBA1 (rabbit polyclonal, 1:250 IF, Wako); and PHF-1 (1:500 IHC and IF, a kind gift from Dr Peter Davies, Bronx, NY), and visualized using appropriate secondary antibody conjugated to an Alexafluor probe (1:200, Lifetechnologies) applied for 1 h. A 0.1% solution of Sudan Black was used to reduce autofluorescence. Slides were coverslipped using Vectashield mounting medium with DAPI (Vector Labs, Burlingame, CA).

### Quantitative image analysis

Three different methods of quantitative image analysis were used in this study: 1) digital positive pixel algorithm, 2) digital nuclear algorithm, 3) and manual counting of IBA1^+^ microglia (only in CA1 region). Briefly, the Aperio ScanScope XT digital slidescanner was used to image the entire stained slide at 40x magnification to create a single high-resolution digital image. The Aperio positive pixel count algorithm (version 9) was used to quantify the amount of specific staining in the region, and the Aperio nuclear algorithm (version 9) was used to determine the number of stained microglia as previously described [16, 17]. The number of IBA1^+^ microglia was counted by morphological appearance in 5 arbitrarily placed 250 × 250 μm boxes in the CA1 region. A researcher (coauthor ADB) blind to all samples’ case histories conducted all data analysis. Immunofluorescence was imaged using a Nikon Eclipse 90i upright microscope equipped with a Nikon DS-Ri1 digital camera.

### Statistics

JMP Software version 10.0 was used for statistical analysis. Normality was assessed using the Shapiro-Wilk test. As there were only a few violations of normality, and ANOVA is robust to such violations [18], a one-way ANOVA followed by a Tukey post hoc analysis was used to compare differences between the five groups. Mean ± SD for quantifications are shown in Table 2. Differences between means were considered significant at *p* < 0.05. Heatmaps were generated using JMP Software version 10.0. All other graphs were generated using GraphPad Prism software version 6.0, with values expressed as mean ± SEM.

**Table 2:** Summary of microglia neuropathological assessment

CD68 positive pixels (Fig. 2)							
	WM	sub	CA1	CA2/3	CA4	DG	hipp ave
NC (N = 9)	7.5 ± 4	2.5 ± 1.6	2.6 ± 2.2	3.2 ± 2.1	2.8 ± 2.2	2.4 ± 1.6	3.5 ± 2.1
HS-aging (N = 7)	8.7 ± 2.4	6 ± 3.5	5.4 ± 2.1	4.1 ± 2.1	3.4 ± 1.2	2.4 ± 1.2	5 ± 1.4
AD (N = 7)	10.6 ± 3.2	6.2 ± 2	5.3 ± 1.5	4.2 ± 2.6	3.5 ± 1.2	4.8 ± 2	5.8 ± 1
AD + HS (N = 4)	8.7 ± 3.6	5.5 ± 2.6	5.8 ± 2.1	3.2 ± 1.2	2.4 ± 1.3	2.5 ± 1.4	4.7 ± 1.2
DLB (N = 12)	7.6 ± 2.8	2.7 ± 1	2.3 ± 0.8	3.4 ± 2.3	2.7 ± 1.1	2.2 ± 0.3	3.5 ± 0.9
CD68 nuclear algorithm (Fig. 4)							
NC (N = 9)	54.7 ± 52.1	13.9 ± 17.9	16 ± 18.5	17 ± 18.3	20 ± 24	10.7 ± 11.3	22.1 ± 22.1
HS-aging (N = 7)	54.8 ± 45.1	34.4 ± 33.6	30.2 ± 20.9	20.4 ± 19.8	19.2 ± 11.9	8.1 ± 7.8	27.8 ± 21.2
AD (N = 7)	100.2 ± 68.4	45.8 ± 35.8	29.2 ± 16.8	16.4 ± 7.5	17.3 ± 13.3	26.3 ± 18.1	39.2 ± 22.4
AD + HS (N = 4)	32.4 ± 15.8	27.2 ± 22.4	28.7 ± 26.6	15.4 ± 14.4	8 ± 6.8	3.8 ± 5.2	19.2 ± 13.7
DLB (N = 12)	53.3 ± 35	11.8 ± 9	11.6 ± 7.7	15.6 ± 11.1	13.3 ± 8.1	8.9 ± 5.9	19.1 ± 10.1
IBA1 nuclear algorithm (Fig. 5)							
NC (N = 9)	101.9 ± 30.2	66.5 ± 14.3	60.8 ± 22.9	74 ± 11.7	82.7 ± 16.8	80.5 ± 18.7	77.7 ± 11.7
HS-aging (N = 7)	143.7 ± 80.8	84.7 ± 39.4	122.1 ± 63.5	142.8 ± 37.3	110.9 ± 50.3	92.3 ± 39.1	116.1 ± 45.4
AD (N = 7)	87.8 ± 55.4	84.1 ± 44.2	64.2 ± 16.2	75.5 ± 19.2	43.2 ± 27.2	50.5 ± 37.2	67.5 ± 26.7
AD + HS (N = 4)	97.9 ± 25.8	116.4 ± 74.9	108.2 ± 42.1	132.4 ± 64.6	94.3 ± 47.1	98.9 ± 47.1	108 ± 41.9
DLB (N = 12)	103.2 ± 55.6	69.3 ± 35.6	70 ± 32	74.2 ± 46.7	83.8 ± 48	74.5 ± 43.9	79.2 ± 40.7
IBA1 positive pixels (Fig. 6)							
NC (N = 9)	2.8 ± 0.8	2.6 ± 0.9	2.4 ± 0.7	3.1 ± 0.8	3.3 ± 1	3.1 ± 0.8	2.9 ± 0.7
HS-aging (N = 7)	4 ± 1.8	2.5 ± 0.9	3.2 ± 1.1	3.9 ± 0.8	3.5 ± 1.3	3.1 ± 1.1	3.3 ± 1
AD (N = 7)	2.7 ± 1.2	2.5 ± 1.1	2.2 ± 0.6	3.1 ± 1	2 ± 1.1	2.1 ± 0.8	2.4 ± 0.7
AD + HS (N = 4)	3.6 ± 0.8	3.4 ± 2.1	3.6 ± 1.1	3.9 ± 1.2	3.1 ± 0.7	3.3 ± 1	3.5 ± 1
DLB (N = 12)	2.8 ± 0.8	1.9 ± 0.6	1.9 ± 0.5	2.3 ± 0.8	2.6 ± 0.9	2.5 ± 0.9	2.3 ± 0.7
Morphological assessment of IBA1^+^ microglia in CA1 region (Fig. 7)							
	ramified	hypertrophic	dystrophic	rod-shaped	amoeboid	total	
NC (N = 9)	16.9 ± 9.8	3.7 ± 7.1	4.4 ± 3.9	2.1 ± 4.1	3.7 ± 5	30.8 ± 10.3	
HS-aging (N = 7)	2.6 ± 2.6	13.8 ± 11.9	32.2 ± 22	2.4 ± 5.1	8.8 ± 8.8	59.9 ± 29.8	
AD (N = 7)	19.7 ± 10.4	4.5 ± 6.1	11.2 ± 9.7	2.6 ± 2.7	4.5 ± 3.6	42.4 ± 11.5	
AD + HS (N = 4)	9.8 ± 8.3	20.2 ± 13.7	19.8 ± 11.2	2.6 ± 3.2	14.6 ± 12.4	67 ± 15.8	
DLB (N = 12)	9.3 ± 6.5	2.5 ± 3.6	13.9 ± 9.3	3.9 ± 4	1.9 ± 2.6	31.3 ± 8.2	

Values represent mean ± SD for the quantification of CD68 and IBA1 immunohistochemistry. Data is plotted in the indicated figures

## Results

Five groups of cases (Table 1) were pathologically-confirmed as either AD (*n* = 7), HS-Aging (*n* = 7), AD + HS-aging (*n* = 4), DLB (n = 12), and NC (*n* = 9). HS-aging and DLB cases were included in this study to determine if there is disease specificity in microglia pathology and to provide the first quantitative analysis of microglia in HS-Aging. Pure HS-aging cases lacked substantial additional pathologies AD-type pathology, or Lewy bodies [19–21], as shown in Table 1. The neuropathological changes associated with neocortical/diffuse Lewy body disease include, by definition, α-synuclein immunoreactive neuronal inclusions (Lewy bodies) and processes in multiple portions of the cerebral neocortex. In pure DLB, there are low levels of amyloid-β pathology or NFTs, as shown in Table 1.

**Fig. 1:**
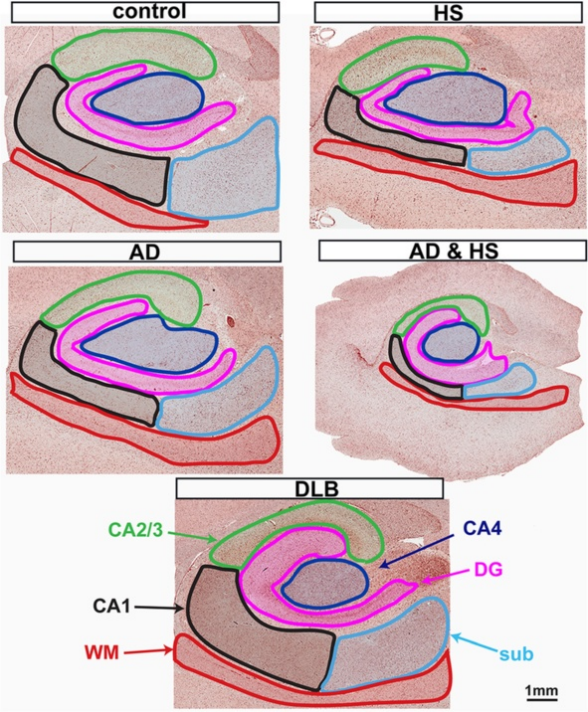
Regions of interest used for microglia analysis. A representative hippocampus is shown for the five neuropathological diagnoses included in this study. The outlines illustrate the boundaries used in identifying the following brain regions: white matter (WM), subiculum (sub), the cornu ammonis (CA) areas, CA1, CA2/3, CA4, and the dentate gyrus (DG). The ROIs shown in the figure are not the actual ROIs used for analysis, as some of the ROIs (WM and sub) could not be included in the image frame, as the brain region was larger than the image frame

Primary goals of this study were to assess regional microglia heterogeneity and to exploit the ability of digital neuropathological quantification to detect in differences microglial morphometry when cases are stratified according to their neurodegenerative diseases. Six regions of interest (ROI) were identified by dividing the hippocampal formation into the dentate gyrus (DG), the cornu ammonis (CA) areas (CA1, CA2/3, and CA4), the subiculum (sub), and the adjacent white matter (WM) (Fig. 1). Representative examples of the ROIs are shown in Fig. 1.

### Pattern of CD68 staining in the hippocampus of autopsy cases

**Fig. 2:**
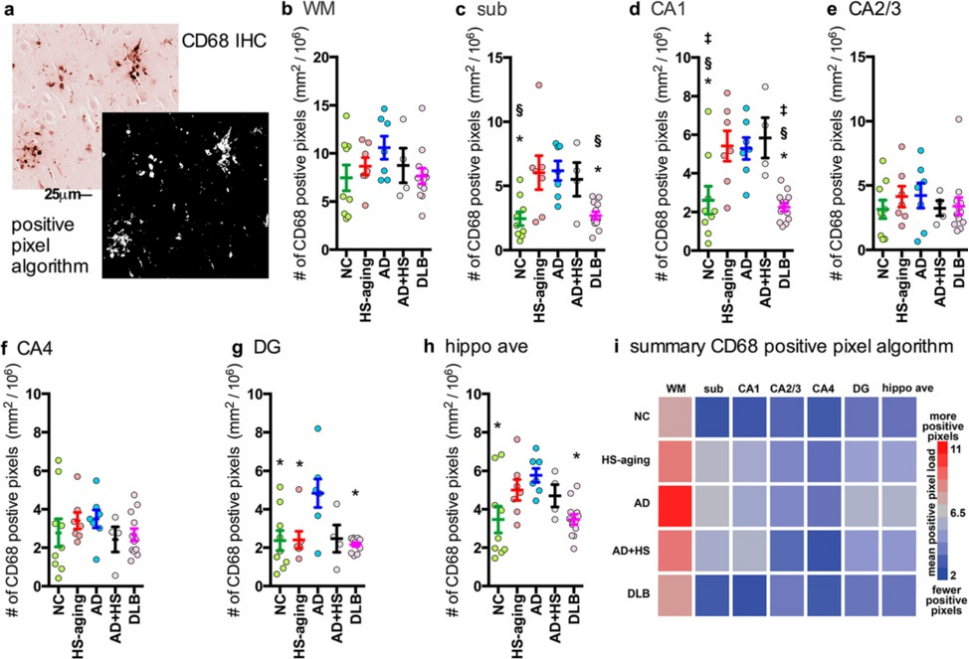
Digital neuropathological quantification using positive pixel algorithm of CD68^+^ immunostaining in the hippocampus of autopsy cases. Representative example of (**a**) CD68 staining and a digitally generated mark-up showing the ability of the positive pixel algorithm to detect the staining. Digital neuropathological quantification of the CD68 staining using the positive pixel algorithm is shown for the (**b**) WM, (**c**) sub, (**d**) CA1, (**e**) CA2/3, (**f**) CA4, (**g**) DG, and for the (**h**) average of the hippocampal formation. Circles represent an individual case, with mean and SEM shown for the group. Statistical comparisons: *p < 0.05 compared to AD cases. ^§^ *p* < 0.05 compared to HS-aging cases. ^‡^ *p* < 0.05 compared to AD + HS-aging cases. (**i**) Heatmap summarizes the results shown in (b-h) (also see Table 2)

**Fig. 3:**
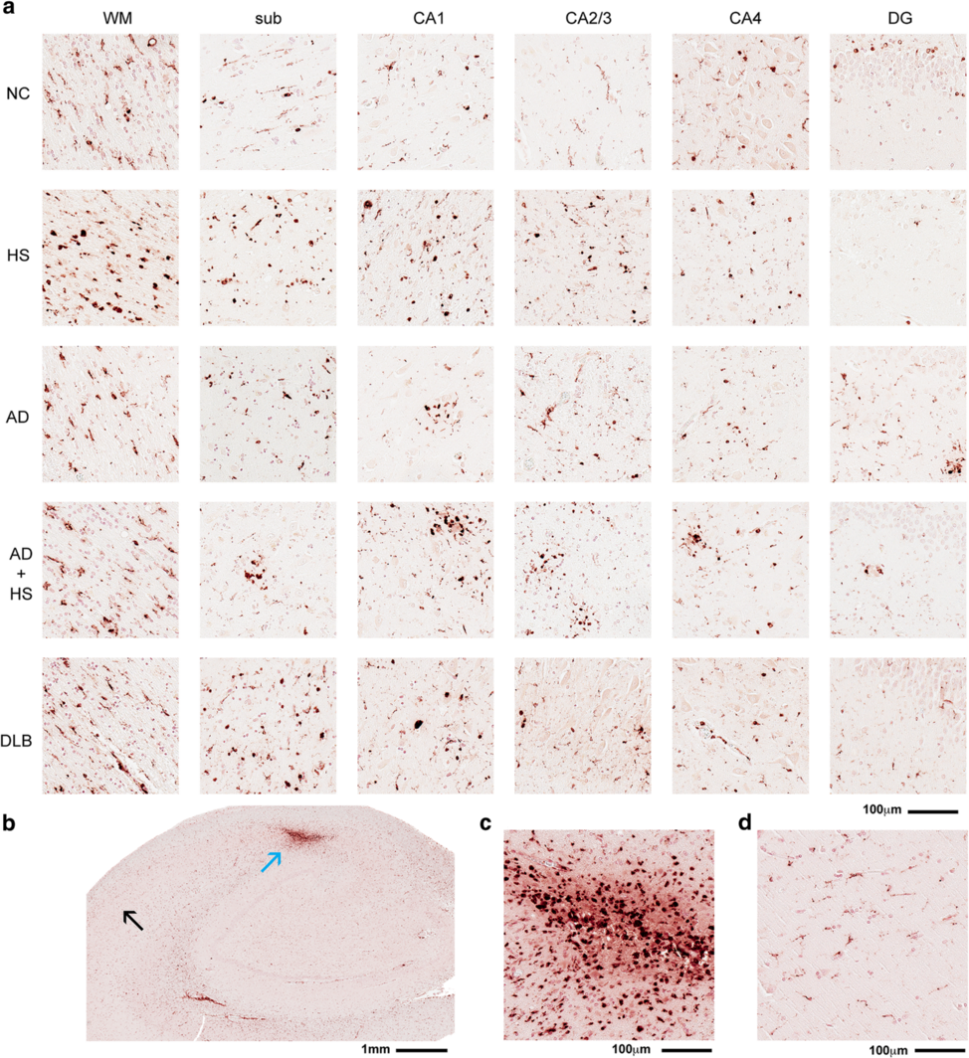
Survey of CD68^+^ staining in the hippocampus of autopsy cases. (**a**) Representative examples of CD68^+^ staining pattern in the brain regions analyzed by digital neuropathological analysis. (**b**) Low power photomicrograph of hippocampus of a DLB individual (case #36) highlights an area of intense staining (*blue arrow*) shown in (**c**), and an area of low CD68 staining (black arrow) in a nearby region (**d**)

**Fig. 4:**
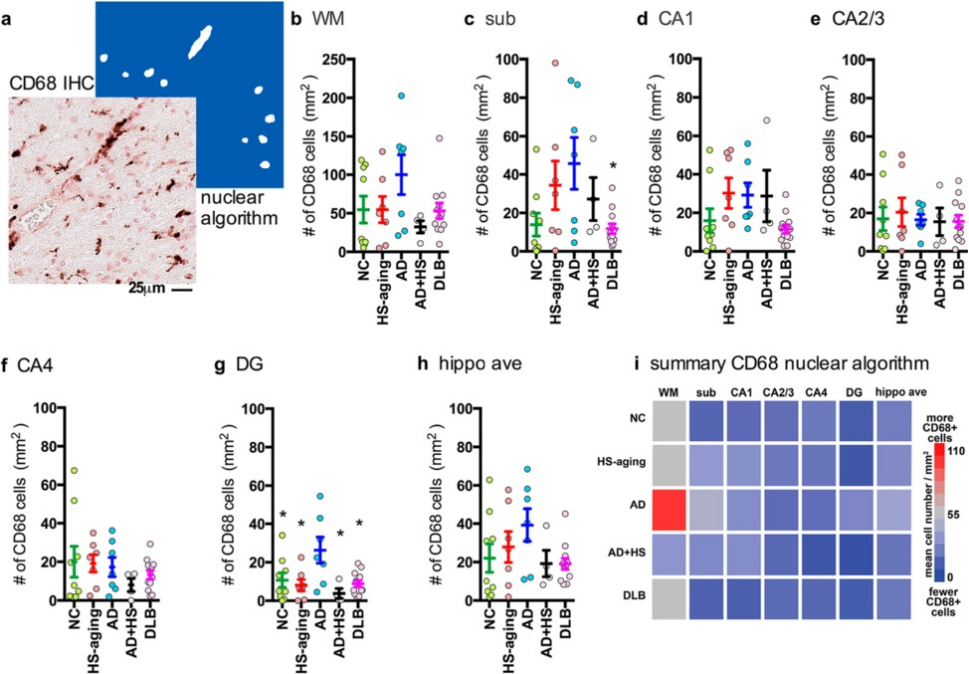
Digital neuropathological quantification using nuclear algorithm of number of large round CD68^+^ cells in the hippocampus of autopsy cases. Representative example of (**a**) CD68 staining and a digitally generated mark-up showing the ability of the nuclear algorithm to detect the staining of the large round cells, but not smaller cells or processes. Digital neuropathological quantification of the CD68 staining using the nuclear algorithm to detect the staining is shown for the (**b**) WM, (**c**) sub (F_4,38_ = 2.9934; *p* = 0.0321), (**d**) CA1, (**e**) CA2/3, (**f**) CA4, (**g**) DG (F_4,38_ = 4.3393; *p* = 0.0061), and for the (**h**) average of the hippocampal formation. Circles represent an individual case, with mean and SEM shown for the group. Statistical comparisons: *p < 0.05 compared to AD cases. (**i**) Heatmap summarizes the results shown in (b-h) (also see Table 2)

Quantification of the CD68 positive pixels is shown in Fig. 2. By a one-way ANOVA a significant effect of disease status was found sub (Fig. 2c; F_4,38_ = 6.3001; *p* = 0.0007), CA1 (Fig. 2d; F_4,38_ = 8.0944; *p* < 0.001), DG (Fig. 2g; F_4,38_ = 5.3332; p = 0.0019), and in the average of the six regions in the hippocampus formation (Fig. 2h; F_4,38_ = 4.3221; p = 0.0062). No significant effect was found by a one-way ANOVA in WM (Fig. 2b), CA2/3, (Fig. 2e), or CA4 (Fig. 2f). HS-aging, AD, and AD + HS-aging were found to have significantly more CD68^+^ staining in the CA1 region compared to NC or DLB cases (Fig. 2d). However, there was no significant difference among the three disease conditions (HS-aging, AD, and AD + HS-aging) in the CA1 region (Fig. 2d). Interestingly, we found significantly more CD68^+^ staining in the DG of AD cases compared to the other four groups (Fig. 2g). When averaged across the six-hippocampal formation sub regions, the AD cases were found to have significantly more CD68^+^ staining compared to NC or DLB groups. Overall, the greatest CD68^+^ staining was seen in the WM, as is evident by the heatmap summary of the CD68 positive pixel analysis (Fig. 2i). A survey of the CD68^+^ staining in the six-hippocampal formation regions illustrates the regional and disease-specific heterogeneity in the staining (Fig. 3). Of note is a large round cell type that can be found in areas of high density staining as shown in Fig. 3b-c. Interestingly, just distal to the very intense accumulation of CD68^+^ cells, the CD68^+^ staining was unremarkable, with a few ramified microglia (Fig. 3d). Quantification of the number of large round CD68^+^ cells was done using the nuclear algorithm, by adjusting the algorithm to detect only the large round cells as shown in Fig. 4. In comparison to design based stereological methods, limitations of the nuclear algorithm include an inability to provide an estimate of the total number of microglia, because of a lack of 3-dimensional volume measurements [22, 23]. Limitations notwithstanding, results of the nuclear algorithm were similar to the positive pixel algorithm, with the HS-aging and AD groups having the greatest number of CD68^+^ cells (Table 2). As shown by the heatmap, the greatest number of CD68^+^ cells was found in the WM of AD cases (Fig. 4).

### Digital quantification of IBA1 staining in the hippocampus of autopsy cases

**Fig. 5:**
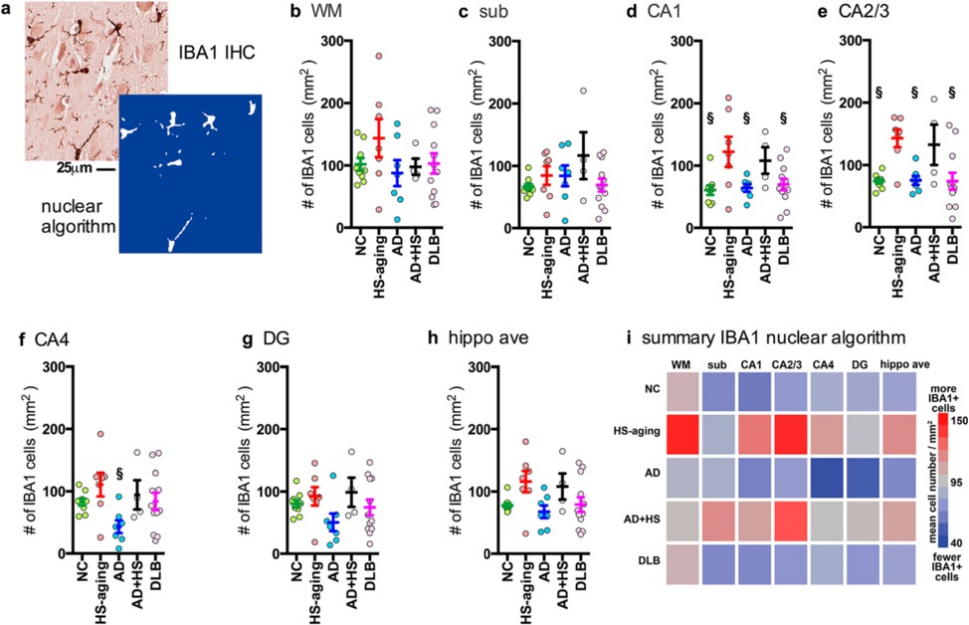
Digital neuropathological quantification using nuclear algorithm of number of IBA1^+^ cells in the hippocampus of autopsy cases. Representative example of (**a**) IBA1 staining and a digitally generated mark-up showing the ability of the nuclear algorithm to detect six stained cells. Digital neuropathological quantification of the IBA1 staining using the nuclear algorithm is shown for the (**b**) WM, (**c**) sub, (**d**) CA1, (**e**) CA2/3, (**f**) CA4, (**g**) DG, and for the (**h**) average of the hippocampal formation.. Circles represent an individual case, with mean and SEM shown for the group. Statistical comparisons: ^§^ *p* < 0.05 compared to HS-aging cases. (**i**) Heatmap summarizes the results shown in (b-g) (also see Table 2)

**Fig. 6:**
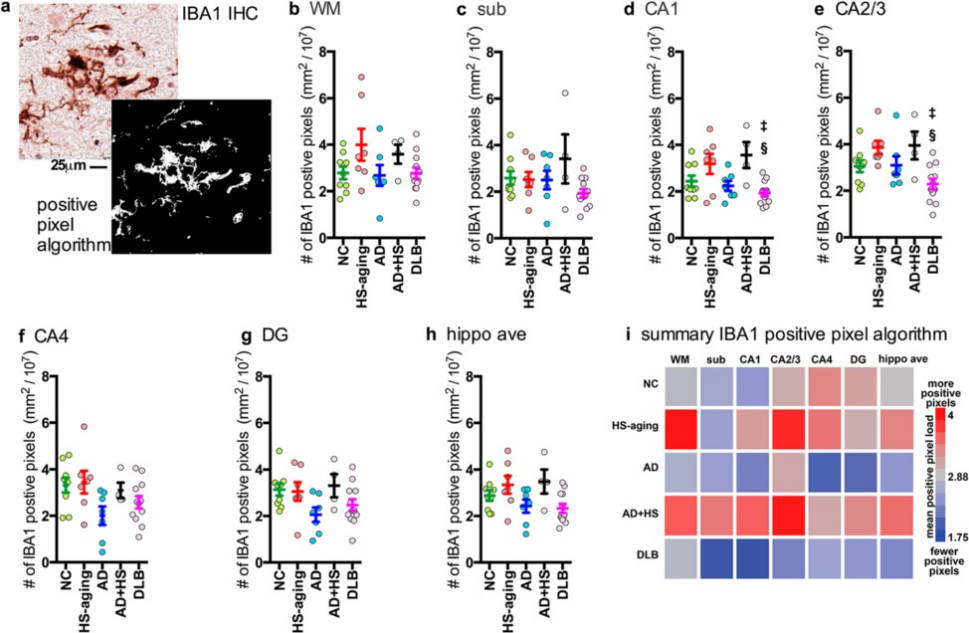
Digital neuropathological quantification using positive pixel algorithm of IBA1^+^ immunostaining in the hippocampus of autopsy cases. Representative example of (a) IBA1 staining and a digitally generated mark-up showing the ability of the positive pixel algorithm to detect the staining. Digital neuropathological quantification of the IBA1 staining using the positive pixel algorithm is shown for the (**b**) WM, (**c**) sub, (**d**) CA1 (F_4,38_ = 5.0943; *p* = 0.0025), (**e**) CA2/3 (F_4,38_ = 4.8888; *p* = 0.0032), (**f**) CA4, (**g**) DG,, and for the (**h**) average of the hippocampal formation (F_4,38_ = 3.0201; *p* = 0.0311). Circles represent an individual case, with mean and SEM shown for the group. Statistical comparisons: *p < 0.05 compared to AD cases. ^§^ *p* < 0.05 compared to HS-aging cases. ^‡^ *p* < 0.05 compared to AD + HS-aging cases. (**i**) Heatmap summarizes the results in (b-h) (also see Table 2)

Quantification of the number of IBA1^+^ cells by the nuclear algorithm is shown in Fig. 5. A representative example of the ability of the algorithm to detect individual cells is shown in Fig. 5a. By a one-way ANOVA, a significant effect of disease status was found in the CA1 region (Fig. 5d; F_4,38_ = 3.9914; *p* = 0.0092), CA2/3 region (Fig. 5e; F_4,38_ = 5.8525; *p* = 0.0011), and in the CA4 (Figure 5f F_4,38_ = 2.6929; *p* = 0.0473). No significant effect was found by a one-way ANOVA in WM (Figure 5b), sub (Fig. 5c), DG (Fig. 5g), or in the average of the six regions in the hippocampus formation (Fig. 5h). In the CA1 region, the HS-aging had an increased number of IBA1^+^ cells compared to NC, AD or DLB. As shown by the heatmap summary, a similar pattern of increased number of IBA1^+^ microglia was found in the HS-aging and AD + HS-aging groups compared to the NC, AD, or DLB groups (Fig. 5i). Quantification of the IBA1 positive pixels (Table 2) also showed a similar pattern of increased IBA1^+^ staining in the HS-aging and AD + HS-aging groups compared to the NC, AD, or DLB groups (Fig. 6).

### IBA1^+^ microglia morphology in the hippocampus of autopsy cases

**Fig. 7:**
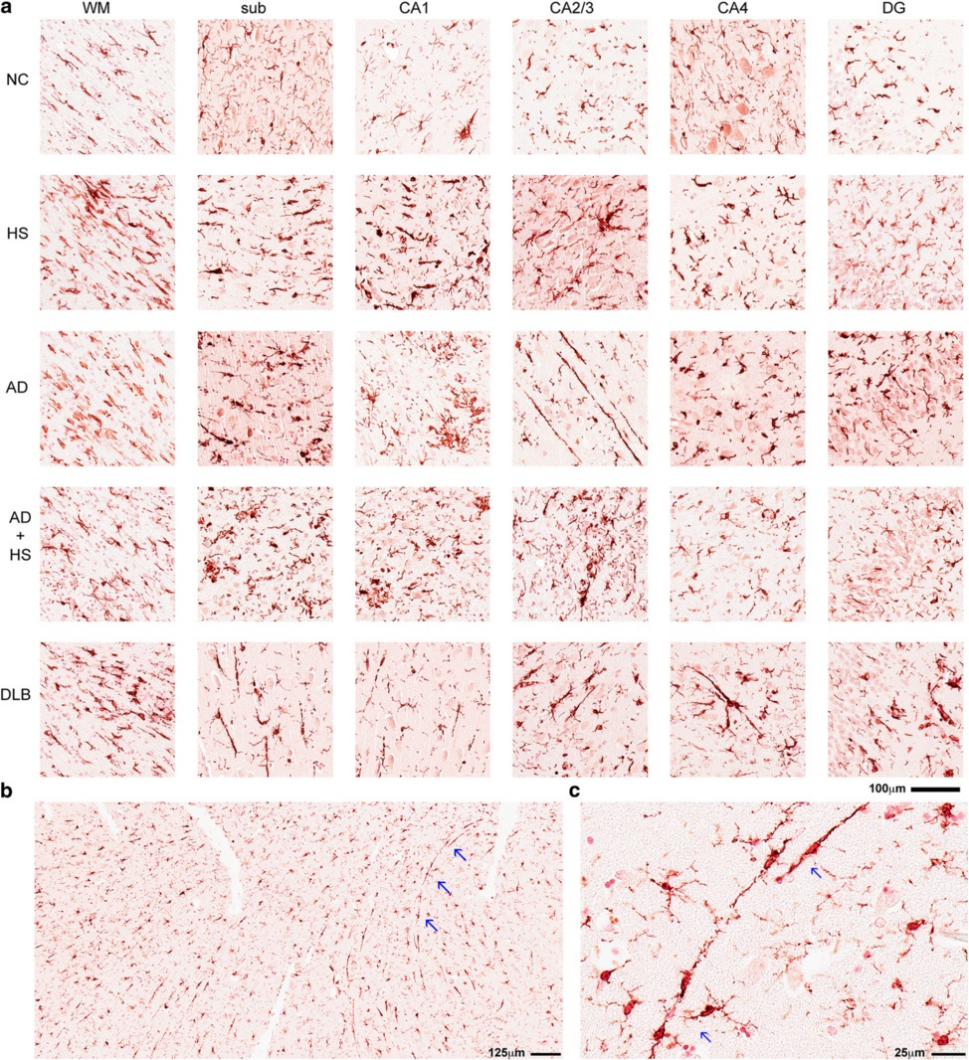
Survey of IBA1^+^ staining in the hippocampus of autopsy cases. (**a**) Representative examples of IBA1^+^ staining pattern in the brain regions analyzed by digital neuropathological analysis(**b**) A low powered photomicrograph shows the widespread distribution of rod shaped microglia in the CA1 region of a DLB individual (case #34). Long trains of microglia (highlighted by blue arrows) are shown at higher magnification in (**c**).

**Fig. 8:**
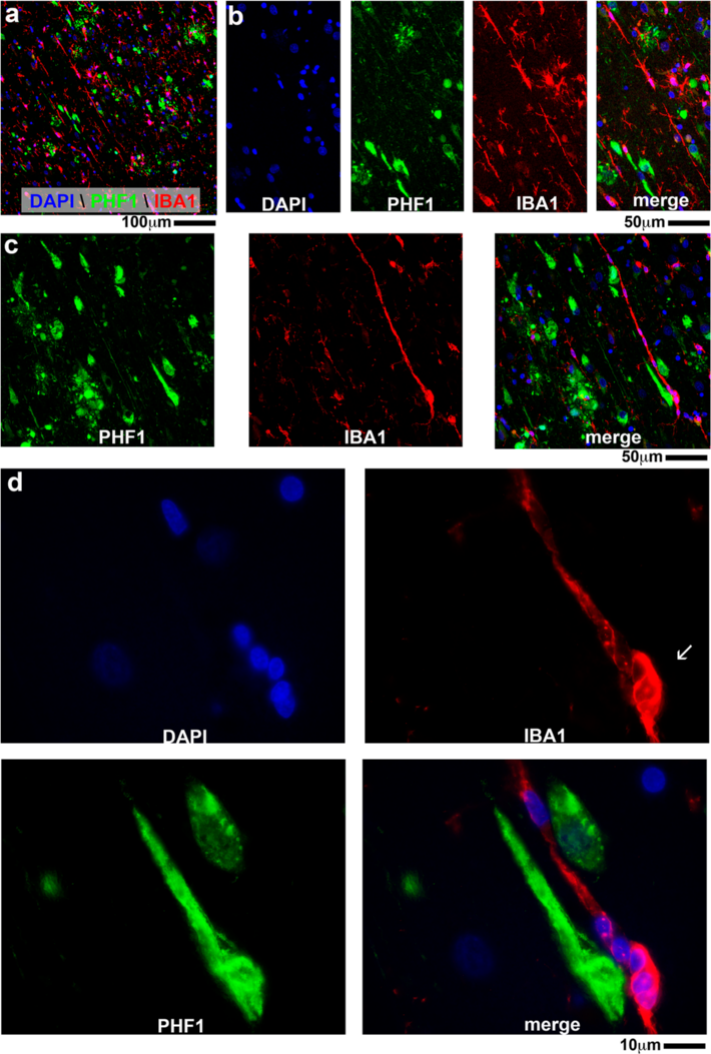
Lack of localization of IBA^+^ rod-shaped microglia to PHF1^+^ neurons in an AD individual (case #23). (**a**) A low powered photomicrograph shows the distribution of rod-shaped microglia next to PHF1^+^ cells. (**b**) A linear group of rod-shaped microglia is shown at a higher magnification. (**c**) A second example of rod-microglia, where the microglia run parallel and between PHF1+ neurons. (**d**) Of note, the polar end of the rod-microglia (*white arrow*) was found to have 5 DAPI^+^ nuclei

An examination (Fig. 7a) of the IBA1^+^ microglia in the six ROIs in the five neuropathologic groups showed remarkable heterogeneity in microglia density, as captured by the digital neuropathological quantification. There was also heterogeneity in IBA1^+^ microglia morphology, which was underappreciated in the digital neuropathological analysis, as microglia density and cell number were measured irrespective of the microglia morphology. For example, a striking pattern of IBA1^+^ microglia morphology is the rod-shaped microglia, which were readily apparent in a subset of cases. As shown in Fig. 7b-c, rod-shaped microglia are characterized by a narrow cell body with a few planar processes. The rod-shaped microglia could be found as individual cells (Fig. 7b), or as long and thin groups of cells that may have fused (Fig. 7b and c and Fig. 8). The appearance of microglia with polarized and parallel processes suggested that the microglia could be following neurites—possibly, degenerating axons or neurons themselves. To test the possibility that microglia could be surrounding degenerating neuronal processes, double label immunofluorescence was performed for microglia (IBA1) and NFTs (PHF1). Fig. 8a shows abundant PHF1^+^ staining and IBA1^+^ rod-shaped microglia in the CA1 region of an AD individual (case #23). We found no evidence of systematic overlap of PHF1^+^ neurites and IBA1^+^ rod-shaped microglia, as shown in Fig. 8b. Rather, long trains of rod-shaped microglia could sometimes be seen to run parallel to and between PHF1^+^ neurons but did not co-localize with the PHF1^+^ staining (Fig. 8c). In this example, the tip of the rod-shaped microglia was near (but not within) a PHF1^+^ structure, and the IBA-1 immunoreactive structure appeared to be a fusion / cluster of multiple cells with 5 clearly visible DAPI^+^ nuclei (Fig. 8d).

**Fig. 9:**
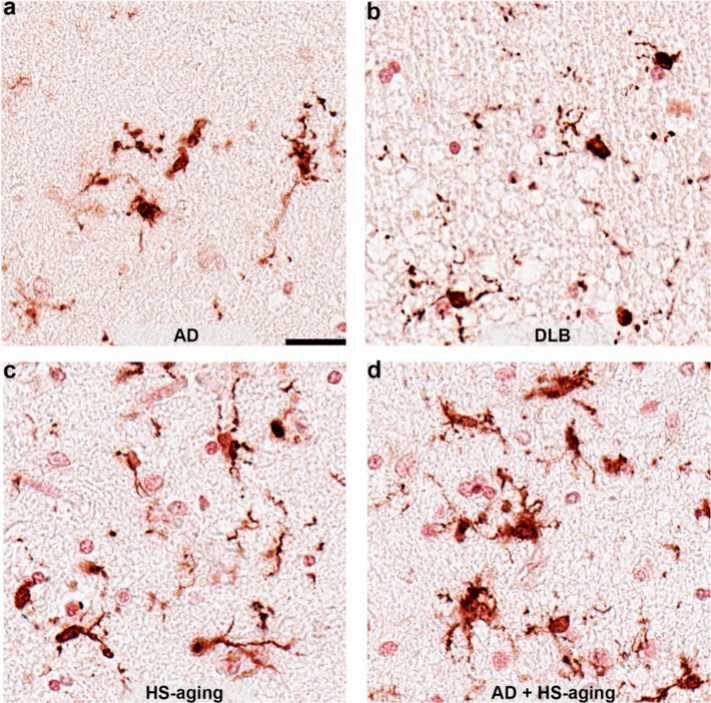
Dystrophic IBA1^+^ microglia in the hippocampus. Examples of IBA1^+^ dystrophic microglia in the CA1 region of AD individual (a; case #20), DLB individual (b; case #33), HS-aging individual (c; case #15), and AD + HS-aging individual (d; case #27). Scale bar is 25 μm

Another pattern of microglia morphology observed was the dystrophic / degenerating microglia, which overlapped morphologically with cells that have been described to have processes that are spheroidal, beaded, de-ramified, or fragmented [24, 25]. Examples of dystrophic / degenerating microglia are shown in Fig. 9. In AD (Fig. 9a) and DLB (Fig. 9b), for example, the dystrophic / degenerating microglia had very thin processes that are beaded and fragmented. In HS-aging (Fig. 9c) and AD + HS-aging (Fig. 9d), dystrophic microglia morphology was more striking, and the processes of the microglia were beaded and tortuous.

**Fig. 10:**
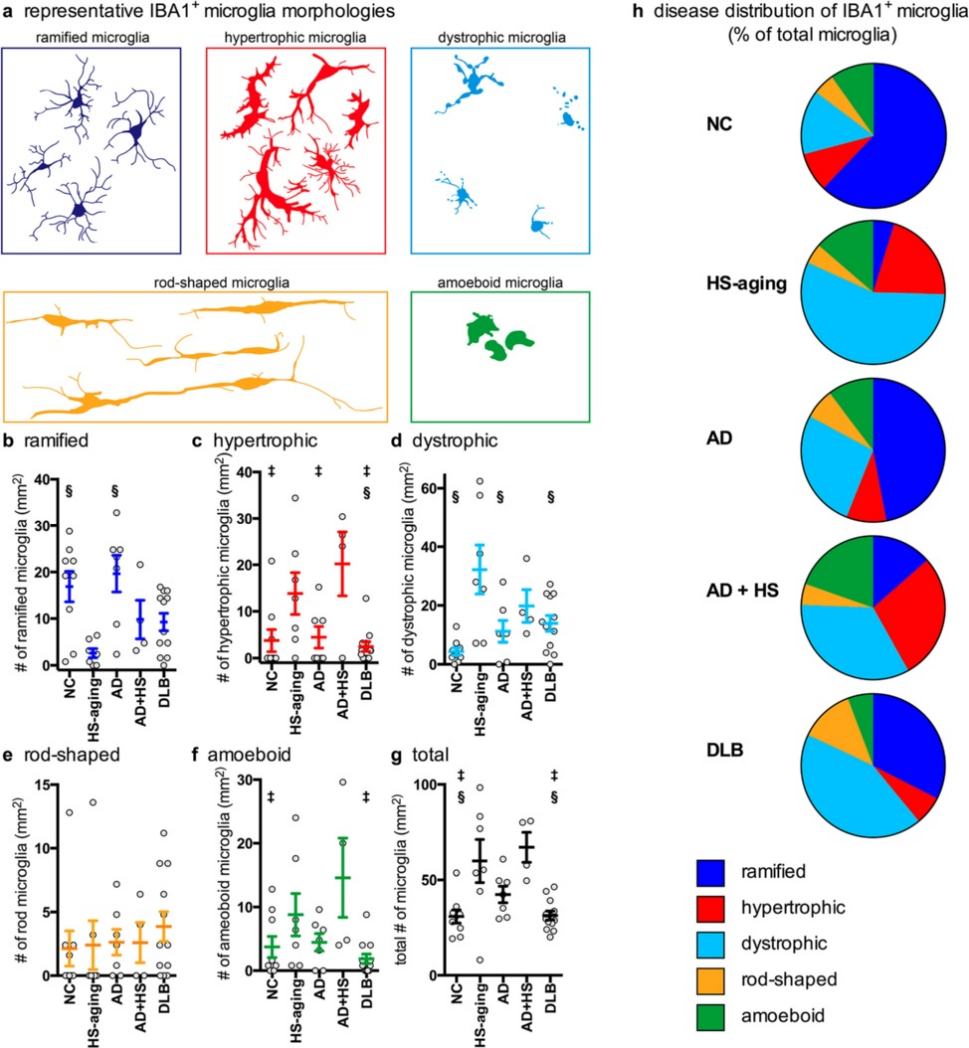
Disease specific patterns in IBA1^+^ microglia morphology. (**a**) Representation of microglia morphologies seen in the hippocampus of aged individuals. The number of microglia was quantified at 40x magnification in five 250 x 250 μm regions of interest (ROIs) that were randomly placed and evenly spaced in the CA1 region. Following the classification shown in (**a**), IBA^+^ microglia were classified as either (**b**) ramified (F_4,38_ = 5.3533; *p* = 0.0019), (**c**) hypertrophic (F_4,38_ = 5.5082; p = 0.0016), (**d**) dystrophic (F_4,38_ = 5.7249; *p* = 0.0012), (**e**) rod-shaped, or (**f**) amoeboid (F_4,38_ = 3.9836; *p* = 0.0093). (**g**) The number of microglia (F_4,38_ = 7.2694; *p* = 0.0002) in the five classifications was summed to get the total number of microglia. The gray circles in (b-g) represent the average number of microglia per mm^2^ for an individual case, with mean and SEM shown for each group (see also Table 2). Statistical comparisons: ^§^ *p* < 0.05 compared to HS-aging cases. ^‡^ *p* < 0.05 compared to AD + HS-aging cases. (**h**) As the total number of microglia significantly varied by group, the number of microglia in each of the five classifications was plotted as a percent of the total number of microglia to illustrate the disease-related patterns in microglia morphology (also see Table 2)

The remarkable diversity in the microglia morphology led us to carefully review and categorize the morphological appearances of the microglia into five distinct classes (Fig. 10a), to allow measurement of changes in the microglia classes associated with the five neurodegenerative disease groups. The five classes of microglia morphologies included: 1) ramified microglia, which have a ‘surveying’ non-reactive microglia morphological appearance, with thin highly branched processes [5, 26]; 2) hypertrophic microglia (often called activated microglia), which have become enlarged, hyper-ramified or may have short thick processes [5, 26]; 3) dystrophic microglia, with processes that are spheroidal, beaded, de-ramified, or fragmented [24–26]; 4) rod-shaped microglia, characterized by a narrow cell body with a few planar processes [27, 26]; and 5) amoeboid microglia, with an enlarged cell body with few to no processes [5, 26]. CD68 staining could clearly identify cells with an amoeboid morphology, and to a lesser extent cells with a ramified morphology. In contrast, IBA1 staining was useful to identify all five microglia morphologies. Therefore, IBA1 staining was used to quantify the distribution in the microglia morphology according to these five subtypes of microglial shapes. Focusing on the CA1 region of the hippocampus, the number of each of the five morphological classes of IBA1^+^ microglia was counted in five randomly placed and evenly distributed 250x250μm regions of interest (ROI). HS-aging cases had fewer ramified microglia than NC (*p* = 0.0091) or AD (*p* = 0.0027) cases (Fig. 10b, Table 2). HS-aging and AD + HS-aging had the most hypertrophic microglia. AD + HS-aging cases had more hypertrophic microglia than NC (*p* = 0.0132), AD (*p* = 0.0270), or DLB (p = 0.0044) cases, and HS-aging cases had more hypertrophic microglia than DLB (*p* = 0.0140) cases (Fig. 10c, Table 2). HS-aging cases had more dystrophic microglia than NC (*p* = 0.0005), AD (*p* = 0.0193), or DLB (*p* = 0.0225) cases (Fig. 10d, Table 2). Quantification of rod-shaped microglia identified a subset of cases with abundant rod-shaped microglia; however, the cases were not specific to a disease group (Fig. 10e, Table 2). AD + HS-aging cases had more amoeboid microglia than NC (*p* = 0.0428), or DLB (*p* = 0.0085) cases (Fig. 10f, Table 2). The total number of microglia in the CA1 region, regardless of morphology, was greatest in HS-aging and AD + HS-aging. AD + HS-aging cases had more total microglia than NC (*p* = 0.046), or DLB (*p* = 0.0035) cases. HS-aging cases had more total microglia than NC (*p* = 0.0072) or DLB (*p* = 0.0048) cases (Fig. 10g, Table 2). As the total number of microglia was found to be altered in the different groups, each of the five microglia classifications was plotted as a percentage of the total number of microglia (Fig. 10h) to help visualize the microglia morphology distributions within and among the different diseases.

## Discussion

The present study underscores the rich diversity of microglial morphologies in the hippocampus of the human brain that may change according to the diseases of aging. We observed regional heterogeneity in the hippocampal formation in the density and number of IBA1^+^ and CD68^+^ microglia. We also observed five morphologically-defined classes of IBA1 labeled microglia: ramified, hypertrophic, dystrophic, rod-shaped, and amoeboid (Fig. 10). Our observations provide evidence for subclasses of microglial morphologies that are seen in particular neurodegenerative diseases. The data provide at least some support for disease-specific microglia pathology in age-related dementias.

A primary goal of our project was to determine if digital neuropathological quantification could detect disease-specific changes in IBA1 and CD68 labeled microglia activation. This is the first study to use digital neuropathological quantification to measure changes in human microglia activation and compare directly the microglia response in the different neurodegenerative diseases, and the first to assess microglia in HS-aging cases. The digital neuropathological quantification was able to detect regional differences in IBA1 and CD68 staining associated with the neuropathological diagnosis. Specifically, we found increased IBA1 and CD68 staining in the HS-aging and AD + HS-aging cases. Interestingly, the spatial pattern and magnitude of the changes in IBA1 and CD68 staining were remarkably similar between the HS-aging and AD + HS-aging cases, suggesting that the HS-aging pattern of microglia staining is dominant over the AD pattern, and that there is not a robust additive effect of the two pathologies. Thus, results of the digital neuropathological quantification clearly show a pattern of microglia activation associated with a specific neurodegenerative disease, but overall the quantification provided only modest sensitivity, with limited diagnostic potential, to separate AD from HS-aging. To determine the reproducibility of the digital quantification, 12 of 39 cases selected at random were replicated in an independent experiment. Even with the modest sample size, a comparison of the number of IBA1 positive pixels in the CA1 regions between the two independent experiments resulted in a R^2^ = 0.7. The results of the replication study support the use of digital neuropathological quantification as a relatively accurate, unbiased, quantitative and efficient means of neuropathological assessment. In the future, development of algorithms that can detect the different microglia phenotypes (Fig. 10) should greatly improve the potential of this approach to detect disease-related changes in microglia morphology, until specific molecular markers that recognize the different microglia morphological states are available.

The long-standing view that microglia become activated and promote neuroinflammation in neurodegenerative disease (toxic gain of function) has been challenged recently by the concept of the dystrophic / diseased microglia (loss of function; see [25, 28]). Support for the hypofunctional (as opposed to activated) microglia model is largely based on morphological examination of IBA1-stained microglia in autopsy samples from aged humans [29–32], as currently there are no specific markers that recognize only degenerating/dystrophic microglia. In addition, the dystrophic microglia phenotype seen in humans is largely absent in rodent models [25]. This may reflect intrinsic differences in human microglia [33], or may reflect limitations in the current animal models. We found that aged individuals without dementia were more likely to have ramified microglia than individuals with dementia (AD, HS-aging, AD + HS-aging, or DLB). Moreover, the present study confirmed that dystrophic microglia are found in aged individuals and in increased numbers in aged individuals with three distinct forms of dementia (AD, HS-aging, and DLB). Our results provide an independent confirmation of the presence of dystrophic microglia described by the Streit laboratory [29–32]. Research at our center has previously shown differences in the M1/M2 microglia phenotype between mild AD and end-stage AD [34], supporting changes in the temporal dynamic of the microglia response to varying degrees of neuropathology. Still, the temporal dynamic of microglia in humans over the course of the lifespan has not been defined and is not fully testable through autopsy (cross-sectional) studies. This is a vital area for future investigation.

The current study highlights the importance of morphology-based readout of cell activity. Rod-shaped microglia are a particularly fascinating microglia phenotype, which was first described by Nissl more than 100 years ago (reviewed in: [27]). Rod-shaped microglia have been described clinically in neurosyphilis, subacute sclerosing panencephalitis, lead encephalopathy, viral encephalitis including HIV-1, and Rasmussen's encephalitis [27, 35]; however, there are few modern reports of rod-shaped microglia in the clinical literature. In experimental models, rod-shaped microglia have been best described following traumatic brain injury [36–38, 16], where a diffuse brain injury will cause the rapid (by 6 h) polarization of microglia to follow along neuronal processes. It has been shown previously in rats that microglia will fuse specifically to the apical dendrite of neurons infected with a retrovirus, but not to un-infected neurons [39]. It is not clear if fusion is occurring in the case of rod-shaped microglia in the current study. Beyond these few reported observations, little is known mechanistically about the chemoattractant signals that drive formation of rod-shaped microglia, or about the specific functions of the rod-shaped microglia in relation to the neuron. We found that rod-shaped microglia could be proximal and parallel to PHF1^+^ neurons/axons, but the rod-shaped microglia did not appear to fuse with or engulf the PHF1^+^ structures. Rod-shaped microglia were present in approximately 60% of cases included in this study, but were most abundant in a subset of cases. Review of the case histories of individuals with abundant rod-shaped microglia did not identify any obvious commonalities. A goal for future studies will be to identify a larger sample of cases with abundant rod-shaped microglia to distinguish clinical-pathological correlations, as a first step in defining mechanistically the functions of this mysterious cell type.

A limitation of this study is the subjective criteria that were used to classify microglia into one of the five morphological categories (Fig. 10). This approach requires an experimenter capable of discerning differences in microglia morphology. The approach also imparts bias and potential for experimenter error. The current study demonstrates that there is a great diversity in the microglia morphology in humans, which is underappreciated, as this diversity is largely absent from animal models. There have been prior attempts to operationalize morphological changes in microglia. A recent study reconstructed microglia from mice and humans using computer-based tracing systems, and was able to provide average cell body size and roundness, along with the number of processes, process length and volume occupied by the processes [40]. That study did not include any samples without neurologic disease, and therefore underestimated the heterogeneity in microglia morphology; for example, they did not describe rod-shaped microglia. Using a similar approach, others have attempted to define classes of microglia morphology, such as the rod-shaped microglia, by calculating cell length to cell width and the number of polar vs. planar branches [36]. Moreover, others have proposed digital 3D reconstruction of the microglia as a means to quantify the microglia morphology [41]. However, before microglia morphological assessment can become standard practice in characterizing the microglia pathology, a consensus must be established on what defines different microglia morphologies, as there is currently no consensus-based agreement on definitions, or terminology for the specific classes of microglia morphology. Our study provides a first step towards this goal and will hopefully provide a framework to move the field forward in this direction.
